# Supplementary material for: Influence of fake news in Twitter during the 2016 US presidential election
Source: Nat Commun. 2019 Jan 2;10:7. doi: 10.1038/s41467-018-07761-2 (PMC6315042; doi:10.1038/s41467-018-07761-2)
Supplement: Supplementary file 2 — Description of Additional Supplementary Files [file 41467_2018_7761_MOESM2_ESM.docx]

Description of Additional Supplementary Files

**Supplementary Data 1:** containing top 10 URLs of each media category along with notes about their classification on fact checking websites (when available), links to the fact checking websites and additional information.
