# Supplementary material for: Influence of fake news in Twitter during the 2016 US presidential election
Source: Nat Commun. 2019 Jan 2;10:7. doi: 10.1038/s41467-018-07761-2 (PMC6315042; doi:10.1038/s41467-018-07761-2)
Supplement: Supplementary file 3 — Supplementary Information [file 41467_2018_7761_MOESM3_ESM.pdf]

Influence of fake news in Twitter during the 2016 US presidential election
Supplementary Information

Bovet et al.

Supplementary Note 1

Breitbart News (extreme bias (right)) is the most dominant media outlet in term of number of tweets among the right end of the outlet categories with 1.8 million tweets (see Supplementary Table 1). Breit-bart is closely aligned with the Trump campaign as Steve Bannon, who co-founded Breitbart, eventually joined Trump's campaign as its chief executive. We also consider separately the websites `shareblue.com` and `bluenationreview.com` in Supplementary Tables 2, 3, 4, 5 & 6 and Supplementary Fig. 1 as they were purchased by David Brock, a political operative of the Hillary Clinton campaign (`https://en.wikipedia.org/wiki/David_Brock`). We examine the relation between `breitbart.com`, `shareblue.com` and `bluenationreview.com` and the rest of with the extremely biased outlets in Supplementary Tables 2, 3, 4, 5 & 6 as well as Supplementary Fig. 1. For this analysis, outlets in the extreme bias (right) news category are split in two sub-categories: Breitbart and the rest of extreme bias (right) news (extreme bias (right)\breitbart). Extreme bias (left) news are also split in two sub-categories: Shareblue + Bluenationreview (SB+BNR) and the rest of extreme bias (left) news (extreme bias (left) \(SB+BNR)). Our analysis re-veals that, although Breitbart represents the largest tweet share of the extreme bias (right) category, the majority (66%) of users sharing links directing toward Breitbart also share links toward other websites of the extreme bias (right) category (Supplementary Table 3). We also find similar characteristics in term of average activity, retweet network structure, activity correlation and causal relations between Breitbart and the rest of the extreme bias (right) category. Removing Breitbart from the extreme bias category and treating it as a separated category does not change our results significantly. Concerning `shareblue.com` and `bluenationreview.com`, we find that they form a minority group of the extreme bias (left) category with a strong overlap (69%) of users with the rest of the extreme bias (left) category and that our results are not changed significantly when we consider them as a separated category.

Supplementary Note 2

We observe the presence of several member of the campaign staffs of each candidate in the top news spreaders. We report the ranking in each news categories of campaign staffers among the top 100 news spreaders in Supplementary Table 9. We see more users linked to the campaign staff of Donald Trump (13) than to the campaign staff of Hillary Clinton (3). We also see that Trump staffers have higher ranks in term of influence and cover a broader spectrum of media categories (fake news (3), extreme bias (right) (9), right (9), right leaning (8), center (8) and left leaning (1)) than Clinton staffers (center (1), left leaning (2), left (1) and extreme bias (left) (1)). This reveals that the Trump team played an important direct role in the diffusion of news in Twitter. Although members of the Trump team are prevalent in the top spreaders of fake, extremely biased (right), right and right leaning news, the causal analysis reveals that they are not driving the activity of Trump

and Clinton supporters which is more importantly influenced by the top center and left leaning spreaders, consisting mainly of journalists. To verify the importance of users linked to the candidates' teams, we repeated the causal analysis after having removed all users linked to the campaigns. We report these results in Supplementary Fig. 4 and Supplementary Table 11. We observe no significant changes in the causal relations between the different groups as the relations are still dominated by center and left leaning top spreaders.

Supplementary Note 3

A possible distinction between the diffusion mechanisms of different news outlets could be due to the fact that some websites aggregates news from other websites instead of producing news. We find four websites that, at least partly, aggregates news: `zerohedge.com` (fake news), `wnd.com` (extreme bias (right)), `realclearpolitics.com` (right leaning) and `truepundit.com` (extreme bias (right)). To understand if the presence of news aggregators in categories other than the center and left leaning could explain the difference in dynamics that we observe, we repeated our analysis of the dynamics after having removed the news aggregators from our dataset. We report the results in Supplementary Tables 12 and 13 and Supplementary Fig. 5. We observe no significant changes in the activity correlations and and that without the news aggregators, the top fake news, extreme bias (right) and right leaning spreaders have a smaller causal effect on the other groups, while the left leaning and center influencers stay the dominant ones. This shows that news aggregators are not responsible for the differences on dynamics that we observe.

	fake news		extreme bias (right) news		right news	
	hostnames	N	hostnames	N	hostnames	N
1	thegatewaypundit.com	761 756	breitbart.com	1 854 920	foxnews.com	1 122 732
2	truthfeed.com	554 955	dailycaller.com	759 504	dailymail.co.uk	474 846
3	infowars.com	478 872	americanthinker.com	179 696	washingtonexaminer.com	462 769
4	therealstrategy.com	241 354	wnd.com	141 336	nypost.com	441 648
5	conservativetribune.com	212 273	freebeacon.com	129 077	bizpacreview.com	170 770
6	zerohedge.com	186 706	newsninja2012.com	127 251	nationalreview.com	164 036
7	rickwells.us	78 736	hannity.com	114 221	lifezette.com	139 257
8	departed.co	72 773	newsmax.com	94 882	redstate.com	105 912
9	thepoliticalinsider.com	66 426	endingthefed.com	88 376	allenbwest.com	104 857
10	therightscoop.com	63 852	truepundit.com	84 967	theconservativetreehouse.com	102 515
11	teaparty.org	48 757	westernjournalism.com	77 717	townhall.com	102 408
12	usapoliticsnow.com	46 252	dailywire.com	67 893	investors.com	102 295
13	clashdaily.com	45 970	newsbusters.org	60 147	theblaze.com	99 029
14	thefederalistpapers.org	45 831	ilovemyfreedom.org	54 772	theamericanmirror.com	91 538
15	redflagnews.com	45 423	100percentfedup.com	54 596	ijr.com	71 558
16	thetruthdivision.com	44 486	pjmedia.com	46 542	judicialwatch.org	70 543
17			weaselzippers.us	45 199	thefederalist.com	55 835
18					hotair.com	55 431
19					conservativereview.com	54 307
20					weeklystandard.com	50 707

	right leaning news		center news		left leaning news	
	hostnames	N	hostnames	N	hostnames	N
1	wsj.com	310 416	cnn.com	2 291 736	nytimes.com	1 811 627
2	washingtontimes.com	208 061	thehill.com	1 200 123	washingtonpost.com	1 640 088
3	rt.com	157 474	politico.com	1 173 717	nbcnews.com	512 056
4	realclearpolitics.com	128 417	usatoday.com	326 198	abcnews.go.com	467 533
5	telegraph.co.uk	82 118	reuters.com	283 962	theguardian.com	439 580
6	forbes.com	64 186	bloomberg.com	266 662	vox.com	369 789
7	fortune.com	57 644	businessinsider.com	239 423	slate.com	279 438
8			apnews.com	198 140	buzzfeed.com	278 642
9			observer.com	128 043	cbsnews.com	232 889
10			fivethirtyeight.com	124 268	politifact.com	198 095
11			bbc.com	118 176	latimes.com	190 994
12			ibtimes.com	72 424	nydailynews.com	188 769
13			bbc.co.uk	71 941	theatlantic.com	177 637
14					mediaite.com	152 877
15					newsweek.com	149 490
16					npr.org	142 143
17					independent.co.uk	127 689
18					cnb.cx	87 094
19					hollywoodreporter.com	84 997

	left news		extreme bias (left) news	
	hostnames	N	hostnames	N
1	huffingtonpost.com	1 057 518	dailynewsbin.com	189 257
2	thedailybeast.com	378 931	bipartisanreport.com	119 857
3	dailykos.com	324 351	bluenationreview.com	75 455
4	rawstory.com	297 256	crooksandliars.com	73 615
5	politicususa.com	293 419	occupydemocrats.com	73 143
6	time.com	252 468	shareblue.com	50 880
7	motherjones.com	210 280	usuncut.com	27 653
8	talkingpointsmemo.com	199 346		
9	msnbc.com	177 090		
10	mashable.com	173 129		
11	salon.com	172 807		
12	thinkprogress.org	172 144		
13	newyorker.com	171 102		
14	mediamatters.org	152 160		
15	nymag.com	121 636		
16	theintercept.com	109 591		
17	thenation.com	54 661		
18	people.com	47 942		

Supplementary Table 1: Hostnames in each media category. We also show the number (N) of tweets with a URL pointing toward each hostname. Tweets with several URLs are counted multiple times.

	N_t	p_t	N_u	p_u	N_t/N_u	$p_{t,n/o}$	$p_{u,n/o}$	$N_{t,n/o}/N_{u,n/o}$
extreme bias (right) news	3 969 639	0.13	294 175	0.07	13.49	0.09	0.03	36.52
breitbart	1 849 871	0.06	163 707	0.04	11.30	0.09	0.04	28.20
extreme bias (right) \breitbart	2 119 876	0.07	238 517	0.05	8.89	0.10	0.03	26.95
extreme bias (left) news	609 503	0.02	99 743	0.02	6.11	0.06	0.03	11.46
SB+BNR	126 191	0.00	28 888	0.01	4.37	0.04	0.03	5.11
extreme bias (left) \(SB+BNR)	483 325	0.02	90 367	0.02	5.35	0.07	0.03	11.37

Supplementary Table 2: Tweet and user volume corresponding to extremely biased news in Twitter. Number, N_t, and proportion, p_t, of tweets with a URL pointing to a website belonging to one of media categories. Number, N_u, and proportion, p_u, of users having sent the corresponding tweets, and average number of tweets per user, N_t/N_u, for each category. Proportion of tweets sent by non-official clients, $p_{t,n/o}$, proportion of users having sent at least one tweet from an non-official client, $p_{u,n/o}$, and average number of tweets per user sent from non-official clients, $N_{t,n/o}/N_{u,n/o}$. The average number of tweets per users and the proportion of tweets sent from unofficial clients are very similar for each sub-categories.

	extreme bias (right)	breitbart	extreme bias (right) \breitbart	extreme bias (left)	SB+BNR	extreme bias (left) \(SB+BNR)
extreme bias (right)	1.00	0.56	0.81	0.06	0.03	0.06
breitbart	0.56	1.00	0.37	0.06	0.02	0.06
extreme bias (right) \breitbart	0.81	0.37	1.00	0.06	0.02	0.06
extreme bias (left)	0.06	0.06	0.06	1.00	0.29	0.91
SB+BNR	0.03	0.02	0.02	0.29	1.00	0.20
extreme bias (left) \(SB+BNR)	0.06	0.06	0.06	0.91	0.20	1.00

Supplementary Table 3: Jaccard indices between the sets of users in the extremely biased news categories. Jaccard indices between the sets of users tweeting URLs directing to extreme bias (right) news outlets, breitbart.com, extreme bias (right) minus breitbart.com (extreme bias (right) \breitbart), extreme bias (left) news outlets, shareblue.com and bluenationreview.com (SB+BNR), extreme bias (left) minus shareblue.com and bluenationreview.com (extreme bias (left) \(SB+BNR)). The Jaccard index between two sets A and B is computed as $J = A \cap B / A \cup B$. Although breitbart represents the largest tweet share of the extreme bias (right) category, the majority (66%) of users sharing links directing toward breitbart also share links toward other websites of the extreme bias (right) category. Shareblue and bluenationreview form a minority group of the extreme bias (left) category with a strong overlap (69%) of users with the rest of the extreme bias (left) category.

	N nodes	N edges	$< k >$	$\sigma(k_{\text{out}})/ < k >$	$\sigma(k_{\text{in}})/ < k >$	$\max(k_{\text{out}})$	$\max(k_{\text{in}})$
extreme bias (right)	249 659	1 637 927	6.56	36 ± 6	2.73 ± 0.03	51 845	588
breitbart	141 924	795 504	5.61	31 ± 6	2.33 ± 0.02	41 039	376
extreme bias (right) \breitbart	201 563	940 161	4.66	43 ± 8	2.28 ± 0.03	51 845	562
extreme bias (left)	78 911	277 483	3.52	33 ± 6	2.49 ± 0.08	23 168	648
SB+BNR	25 956	59 515	2.29	45 ± 6	1.34 ± 0.01	15 544	65
extreme bias (left) \(SB+BNR)	70 405	223 532	3.17	31 ± 8	2.4 ± 0.1	23 168	648

Supplementary Table 4: Retweet networks characteristics for extremely biased news categories. We show the number of nodes and edges (links) of the networks, the average degree, $\langle k \rangle = \langle k_{\text{in}} \rangle = \langle k_{\text{out}} \rangle$, (the in-/out-degree of a node is the number of in-going/out-going links attached to it). The out-degree of a node, i.e. a user, is equal to the number of different users that have retweeted at least one of her/his tweets. Its in-degree represents the number of different users she/he retweeted. The ratio of the standard deviation and the average of the in- and out-degree distribution, $\sigma(k_{\text{in}})/\langle k \rangle$ and $\sigma(k_{\text{out}})/\langle k \rangle$, measures the heterogeneity of the connectivity of each networks. As the standard deviation of heavy-tailed degree distributions can depend on the network size, we computed the values of $\sigma(k_{\text{in}})/\langle k \rangle$ and $\sigma(k_{\text{out}})/\langle k \rangle$ with a bootstrap procedure. The average degree and the heterogeneity of the degree distributions are similar for each sub-categories.

	fake news	breitbart	extreme bias (right) \breitbart	right	right leaning	pro-Trump	center	left leaning	left	SB+BNR	extreme bias (left) \(SB+BNR)	pro-Clinton
fake news	1.00	0.40	0.44	0.49	0.41	0.54	0.34	0.34	0.39	0.13	0.30	0.34
breitbart	0.40	1.00	0.36	0.35	0.28	0.40	0.27	0.30	0.32	0.11	0.28	0.29
extreme bias (right) \breitbart	0.44	0.36	1.00	0.49	0.29	0.47	0.28	0.28	0.35	0.11	0.26	0.27
right	0.49	0.35	0.49	1.00	0.37	0.57	0.37	0.37	0.42	0.12	0.33	0.36
right leaning	0.41	0.28	0.29	0.37	1.00	0.42	0.36	0.32	0.35	0.15	0.23	0.36
pro-Trump	0.54	0.40	0.47	0.57	0.42	1.00	0.58	0.61	0.59	0.18	0.39	0.73
center	0.34	0.27	0.28	0.37	0.36	0.58	1.00	0.60	0.55	0.20	0.30	0.65
left leaning	0.34	0.30	0.28	0.37	0.32	0.61	0.60	1.00	0.63	0.23	0.36	0.73
left	0.39	0.32	0.35	0.42	0.35	0.59	0.55	0.63	1.00	0.20	0.38	0.68
SB+BNR	0.13	0.11	0.11	0.12	0.15	0.18	0.20	0.23	0.20	1.00	0.15	0.20
extreme bias (left) \(SB+BNR)	0.30	0.28	0.26	0.33	0.23	0.39	0.30	0.36	0.38	0.15	1.00	0.35
pro-Clinton	0.34	0.29	0.27	0.36	0.36	0.73	0.65	0.73	0.68	0.20	0.35	1.00

Supplementary Table 5: Pearson correlation coefficient between the activity corresponding to different media categories. The correlation profile of breitbart and extreme bias (left) minus breitbart are very similar. Extreme bias (left) minus breitbart has a slightly higher correlation with the right new and with the pro-Trump supporters than breitbart alone. SB+BNR has a relatively different correlation profile than extreme bias (left) minus SB+BNR, as it is poorly correlated with all of other categories.

↙	pro-Clinton	pro-Trump	fake news	breitbart	extreme bias (right) \breitbart	right
pro-Clinton	0.65 ± 0.01	0.14 ± 0.01	0.007 ± 0.008	0.0004 ± 0.0003	0.0008 ± 0.0010	0.005 ± 0.007
pro-Trump	0.13 ± 0.02	0.45 ± 0.01	0.004 ± 0.005	0.000 ± 0.001	0.0003 ± 0.0004	0.002 ± 0.005
fake news	0.021 ± 0.004	0.10 ± 0.01	0.15 ± 0.01	0.02 ± 0.01	0.06 ± 0.01	0.01 ± 0.01
breitbart	0.05 ± 0.01	0.06 ± 0.01	0.03 ± 0.01	0.20 ± 0.02	0.05 ± 0.01	0.02 ± 0.01
extreme bias (right) \breitbart	0.015 ± 0.009	0.005 ± 0.002	0.01 ± 0.01	0.04 ± 0.01	0.23 ± 0.01	0.05 ± 0.01
right	0.019 ± 0.008	0.027 ± 0.009	0.03 ± 0.01	0.03 ± 0.02	0.04 ± 0.01	0.17 ± 0.01
right leaning	0.016 ± 0.008	0.020 ± 0.009	0.02 ± 0.01	0.01 ± 0.02	0.03 ± 0.01	0.06 ± 0.01
center	0.03 ± 0.01	0.011 ± 0.006	0.022 ± 0.007	0.0017 ± 0.0007	0.0024 ± 0.0008	0.011 ± 0.007
left leaning	0.04 ± 0.01	0.007 ± 0.003	0.004 ± 0.002	0.0024 ± 0.0008	0.0023 ± 0.0008	0.011 ± 0.007
left	0.03 ± 0.01	0.03 ± 0.01	0.010 ± 0.008	0.0024 ± 0.0010	0.0031 ± 0.0009	0.009 ± 0.008
(SB+BNR)	0.09 ± 0.02	0.012 ± 0.002	0.023 ± 0.008	0.025 ± 0.008	0.03 ± 0.01	0.003 ± 0.001
extreme bias (left) \(SB+BNR)	0.09 ± 0.02	0.03 ± 0.01	0.02 ± 0.01	0.04 ± 0.01	0.027 ± 0.008	0.003 ± 0.001

↙	right leaning	center	left leaning	left	(SB+BNR)	extreme bias (left) \(SB+BNR)
pro-Clinton	0.001 ± 0.001	0.046 ± 0.007	0.063 ± 0.008	0.04 ± 0.01	0.037 ± 0.009	0.016 ± 0.006
pro-Trump	0.0005 ± 0.0007	0.037 ± 0.008	0.034 ± 0.007	0.020 ± 0.007	0.008 ± 0.003	0.008 ± 0.005
fake news	0.06 ± 0.01	0.026 ± 0.010	0.015 ± 0.003	0.013 ± 0.009	0.01 ± 0.01	0.02 ± 0.01
breitbart	0.04 ± 0.01	0.042 ± 0.009	0.019 ± 0.002	0.004 ± 0.001	0.003 ± 0.001	0.042 ± 0.009
extreme bias (right) \breitbart	0.06 ± 0.01	0.045 ± 0.009	0.030 ± 0.010	0.029 ± 0.009	0.03 ± 0.01	0.010 ± 0.009
right	0.09 ± 0.01	0.043 ± 0.009	0.017 ± 0.003	0.0034 ± 0.0010	0.035 ± 0.008	0.002 ± 0.001
right leaning	0.22 ± 0.01	0.044 ± 0.009	0.034 ± 0.009	0.0036 ± 0.0009	0.026 ± 0.008	0.0029 ± 0.0008
center	0.009 ± 0.009	0.266 ± 0.009	0.18 ± 0.01	0.032 ± 0.009	0.014 ± 0.002	0.030 ± 0.009
left leaning	0.003 ± 0.002	0.17 ± 0.01	0.291 ± 0.009	0.039 ± 0.010	0.043 ± 0.009	0.028 ± 0.008
left	0.02 ± 0.01	0.08 ± 0.01	0.10 ± 0.01	0.16 ± 0.01	0.02 ± 0.01	0.06 ± 0.01
(SB+BNR)	0.003 ± 0.001	0.028 ± 0.009	0.045 ± 0.010	0.03 ± 0.01	0.21 ± 0.01	0.06 ± 0.01
extreme bias (left) \(SB+BNR)	0.02 ± 0.01	0.026 ± 0.010	0.045 ± 0.008	0.034 ± 0.010	0.022 ± 0.009	0.25 ± 0.01

Supplementary Table 6: Maximum causal effect with Breitbart and SB+BNR separated. Maximum causal effect values (± s.d.) between the activity of the top 100 spreaders of each media category and the candidate supporters when considering Breitbart and shareblue+bluenationreview as separated from extreme bias (right) and extreme bias (left), respectively.

	N nodes	N edges	$< k >$	$\sigma(k_{\text{out}})/ < k >$	$\sigma(k_{\text{in}})/ < k >$	$\max(k_{\text{out}})$	$\max(k_{\text{in}})$
fake news	175 605	1 854 439	10.56	47 ± 7	3.18 ± 0.06	104 840	1861
extreme bias (right)	249 659	2 699 930	10.81	56 ± 12	3.55 ± 0.06	172 769	1712
right	345 644	2 799 298	8.10	63 ± 20	3.57 ± 0.08	243 101	1998
right leaning	216 026	611 563	2.83	55 ± 14	2.33 ± 0.08	53 248	468
center	864 733	4 140 477	4.79	94 ± 55	4.7 ± 0.6	680 126	5703
left leaning	1 043 436	4 965 956	4.76	75 ± 27	4.9 ± 0.3	279 049	2547
left	536 903	2 707 064	5.04	65 ± 17	5.0 ± 0.2	119 444	1830
extreme bias (left)	78 911	426 452	5.40	52 ± 9	3.27 ± 0.08	50 415	1003

Supplementary Table 7: Weighted retweet networks characteristics. We show the number of nodes and edges (links) of the networks, the average degree, $\langle k \rangle = \langle k_{\text{in}} \rangle = \langle k_{\text{out}} \rangle$, (the in-/out-degree of a node is the number of in-going/out-going links attached to it). Here, the weight of a link represents the number of retweets from a user to another. In a directed network, the average in-degree and out-degree are always equal. The out-degree of a node, i.e. a user, is equal to the number of times other users have retweeted her/his tweets. Its in-degree represents the number of times she/he retweeted other users. The ratio of the standard deviation and the average of the in- and out-degree distribution, $\sigma(k_{\text{in}})/\langle k \rangle$ and $\sigma(k_{\text{out}})/\langle k \rangle$, measures the heterogeneity of the connectivity of each networks. As the standard deviation of heavy-tailed degree distributions can depend on the network size, we computed the values of $\sigma(k_{\text{in}})/\langle k \rangle$ and $\sigma(k_{\text{out}})/\langle k \rangle$ with a bootstrap procedure.

	fake news	fake (no aggr.)	breitbart	extreme bias (right)	extreme bias (right no aggr.)	extreme bias (right) \breitbart	right	right leaning	right leaning (no aggr.)	center	left leaning	left	extreme bias (left)	extreme bias (left) \(SB+BNR)	SB+ BNR
fake news	100	96	44	40	40	37	31	24	20	10	3	0	0	0	0
fake (no aggr.)	96	100	45	41	41	38	30	23	20	10	3	0	0	0	0
breitbart	44	45	100	73	76	46	40	33	27	15	3	0	0	0	0
extreme bias (right)	40	41	73	100	96	72	43	35	29	16	3	0	0	0	0
extreme bias (right no aggr.)	40	41	76	96	100	70	44	36	30	17	3	0	0	0	0
extreme bias (right) \breitbart	37	38	46	72	70	100	39	30	28	16	3	0	0	0	0
right	31	30	40	43	44	39	100	36	31	19	3	0	0	0	0
right leaning	24	23	33	35	36	30	36	100	82	22	4	2	0	0	0
right leaning (no aggr.)	20	20	27	29	30	28	31	82	100	23	5	3	1	0	1
center	10	10	15	16	17	16	19	22	23	100	18	9	1	0	2
left leaning	3	3	3	3	3	3	3	4	5	18	100	14	1	0	2
left	0	0	0	0	0	0	0	2	3	9	14	100	16	14	13
extreme bias (left)	0	0	0	0	0	0	0	0	1	1	1	16	100	81	42
extreme bias (left) \(SB+BNR)	0	0	0	0	0	0	0	0	0	0	0	14	81	100	26
SB+BNR	0	0	0	0	0	0	0	0	1	2	2	13	42	26	100

Supplementary Table 8: Intersection between sets of the top 100 news spreaders from each media category. We observe that the set of top 100 influencers does not change greatly when removing the news aggregators. The sets of top 100 fake news and fake news without aggregators influencers have 96 influencers in common. Their are also 96 influencers in common in the top 100 sets of extreme bias (right) and extreme bias (right) without aggregators. The right leaning and right leaning without aggregators top 100 influencers see the largest change, but still have 82 influencers in common.

	fake news fake	extreme bias (right)	right	lean right	center	lean left	left	extreme bias (left)
@realDonaldTrump (T)	5	1	2	4	28	53		
@DonaldJTrumpJr (T)	14	12	25	62	84			
@DanScavino (T)	73	20	36	16	76			
@BreitbartNews (T)		3						
@EricTrump (T)		45	31					
@TeamTrump (T)		16	17	9	34			
@PaulManafort (T)		59	45	17	82			
@KellyannePolls (T)		19	13	8	20			
@JasonMillerinDC (T)		60	26	15	43			
@seanspicer (T)			80	38	83			
@RealBenCarson (T)								
@BreitbartXM (T)		65						
@BreitbartTech (T)		87						
@HillaryClinton (C)					67	17	51	
@JesseLehrich (C)						85		
@Shareblue (C)								6

Supplementary Table 9: Collective influence ranking of Twitter users linked to the campaign staffs. Influence ranking of users in the campaign staffs of Donald Trump (T) and Hillary Clinton (C) among the top 100 news spreaders of each media category. Based on `http://www.p2016.org/trump/trumporggen.html` and `http://www.p2016.org/clinton/clintonorggen.html`. We consider accounts related to Breitbart.com to be linked to the Trump team because of Steve Bannon who co-founded Breitbart and was chief executive of Donald Trump's presidential campaign (`https://en.wikipedia.org/wiki/Steve_Bannon`). We consider @Shareblue to be linked to Clinton team because of David Brock, a political operative of the Hillary Clinton campaign who purchased Shareblue (`https://en.wikipedia.org/wiki/David_Brock`).

	fake news	extreme bias (right)	right	right leaning	center	left leaning	left	extreme bias (left)	pro-Trump	pro-Clinton
fake news	1.00	0.50	0.49	0.41	0.34	0.34	0.39	0.30	0.54	0.34
extreme bias (right)	0.50	1.00	0.52	0.34	0.34	0.36	0.41	0.32	0.53	0.34
right	0.49	0.52	1.00	0.37	0.37	0.37	0.42	0.32	0.57	0.36
right leaning	0.41	0.34	0.37	1.00	0.36	0.32	0.35	0.26	0.42	0.36
center	0.34	0.34	0.37	0.36	1.00	0.60	0.55	0.34	0.58	0.65
left leaning	0.34	0.36	0.37	0.32	0.60	1.00	0.63	0.40	0.61	0.73
left	0.39	0.41	0.42	0.35	0.55	0.63	1.00	0.41	0.59	0.68
extreme bias (left)	0.30	0.32	0.32	0.26	0.34	0.40	0.41	1.00	0.41	0.38
pro-Trump	0.54	0.53	0.57	0.42	0.58	0.61	0.59	0.41	1.00	0.73
pro-Clinton	0.34	0.34	0.36	0.36	0.65	0.73	0.68	0.38	0.73	1.00

Supplementary Table 10: Pearson correlation coefficient between the activity corresponding to each media categories.

↙	pro-Clinton	pro-Trump	fake news no staff	extreme bias (right) no staff	right no staff
pro-Clinton	0.65 ± 0.01	0.14 ± 0.01	0.003 ± 0.006	0.010 ± 0.008	0.011 ± 0.008
pro-Trump	0.12 ± 0.02	0.45 ± 0.01	0.001 ± 0.002	0.0010 ± 0.0006	0.002 ± 0.001
fake news	0.018 ± 0.003	0.10 ± 0.01	0.15 ± 0.01	0.04 ± 0.02	0.03 ± 0.01
extreme bias (right)	0.03 ± 0.01	0.008 ± 0.003	0.03 ± 0.01	0.18 ± 0.02	0.047 ± 0.010
right	0.009 ± 0.002	0.006 ± 0.002	0.03 ± 0.01	0.04 ± 0.01	0.20 ± 0.01
right leaning	0.019 ± 0.008	0.040 ± 0.008	0.02 ± 0.01	0.02 ± 0.01	0.08 ± 0.01
center	0.03 ± 0.01	0.013 ± 0.009	0.012 ± 0.008	0.0010 ± 0.0006	0.007 ± 0.010
left leaning	0.04 ± 0.01	0.008 ± 0.005	0.002 ± 0.002	0.0006 ± 0.0005	0.008 ± 0.008
left	0.07 ± 0.02	0.02 ± 0.01	0.019 ± 0.009	0.0026 ± 0.0009	0.003 ± 0.002
extreme bias (left)	0.07 ± 0.02	0.02 ± 0.01	0.03 ± 0.01	0.03 ± 0.01	0.004 ± 0.001

↙	right leaning no staff	center no staff	left leaning no staff	left no staff	extreme bias (left) no staff
pro-Clinton	0.0009 ± 0.0010	0.054 ± 0.008	0.071 ± 0.008	0.013 ± 0.009	0.017 ± 0.008
pro-Trump	0.0005 ± 0.0005	0.016 ± 0.003	0.036 ± 0.007	0.027 ± 0.008	0.013 ± 0.006
fake news	0.06 ± 0.01	0.026 ± 0.009	0.014 ± 0.002	0.005 ± 0.001	0.029 ± 0.008
extreme bias (right)	0.06 ± 0.01	0.039 ± 0.009	0.019 ± 0.003	0.030 ± 0.009	0.033 ± 0.008
right	0.09 ± 0.01	0.043 ± 0.009	0.018 ± 0.003	0.031 ± 0.009	0.033 ± 0.008
right leaning	0.23 ± 0.01	0.036 ± 0.009	0.031 ± 0.010	0.0032 ± 0.0010	0.0025 ± 0.0007
center	0.004 ± 0.009	0.261 ± 0.010	0.17 ± 0.01	0.018 ± 0.008	0.005 ± 0.009
left leaning	0.002 ± 0.002	0.138 ± 0.010	0.313 ± 0.009	0.015 ± 0.008	0.001 ± 0.001
left	0.016 ± 0.008	0.07 ± 0.01	0.10 ± 0.01	0.16 ± 0.01	0.06 ± 0.01
extreme bias (left)	0.02 ± 0.01	0.023 ± 0.003	0.051 ± 0.008	0.03 ± 0.01	0.26 ± 0.01

Supplementary Table 11: Maximum causal effects without campaign staffers. Maximum causal effect values (± s.d.) between the activity of the top 100 spreaders of each media category, where member of the staff of each candidate campaign (see Supplementary Table 9) are removed, and the activity of the presidential candidate supporters.

	fake news (no aggregators)	extreme bias (right) (no aggregators)	right	right leaning (no aggregators)	center	left leaning	left	extreme bias (left)	pro-Trump	pro-Clinton
fake (no aggregators)	1.00	0.49	0.48	0.39	0.33	0.34	0.38	0.29	0.53	0.33
extreme bias (right) (no aggregators)	0.49	1.00	0.52	0.32	0.34	0.35	0.41	0.31	0.52	0.33
right	0.48	0.52	1.00	0.35	0.37	0.37	0.42	0.32	0.57	0.36
right leaning (no aggregators)	0.39	0.32	0.35	1.00	0.35	0.31	0.34	0.25	0.41	0.35
center	0.33	0.34	0.37	0.35	1.00	0.60	0.55	0.34	0.58	0.65
left leaning	0.34	0.35	0.37	0.31	0.60	1.00	0.63	0.40	0.61	0.73
left	0.38	0.41	0.42	0.34	0.55	0.63	1.00	0.41	0.59	0.68
extreme bias (left)	0.29	0.31	0.32	0.25	0.34	0.40	0.41	1.00	0.41	0.38
pro-Trump	0.53	0.52	0.57	0.41	0.58	0.61	0.59	0.41	1.00	0.73
pro-Clinton	0.33	0.33	0.36	0.35	0.65	0.73	0.68	0.38	0.73	1.00

Supplementary Table 12: Pearson correlation coefficient between the activity corresponding to each media categories without the news aggregators. We observe no significant changes in the correlation coefficients between the analysis with (Tab. 10) and without news aggregators. The maximum difference in correlation (0.02) is between the right leaning and extreme bias (right).

↙	pro-Clinton	pro-Trump	fake news (no aggr.)	extreme bias (right, no aggr.)	right
pro-Clinton	0.65 ± 0.01	0.14 ± 0.01	0.015 ± 0.005	0.0014 ± 0.0005	0.003 ± 0.003
pro-Trump	0.13 ± 0.02	0.45 ± 0.01	0.010 ± 0.006	0.0011 ± 0.0005	0.0010 ± 0.0005
fake news (no aggr.)	0.05 ± 0.01	0.10 ± 0.01	0.14 ± 0.01	0.09 ± 0.01	0.02 ± 0.01
extreme bias (right) (no aggr.)	0.03 ± 0.01	0.005 ± 0.003	0.03 ± 0.01	0.20 ± 0.01	0.05 ± 0.01
right	0.023 ± 0.008	0.04 ± 0.01	0.02 ± 0.01	0.03 ± 0.01	0.19 ± 0.01
right leaning (no aggr.)	0.006 ± 0.002	0.002 ± 0.002	0.02 ± 0.01	0.012 ± 0.010	0.05 ± 0.01
center	0.04 ± 0.01	0.026 ± 0.010	0.012 ± 0.007	0.0012 ± 0.0007	0.015 ± 0.008
left leaning	0.04 ± 0.01	0.016 ± 0.005	0.003 ± 0.001	0.0006 ± 0.0004	0.011 ± 0.007
left	0.06 ± 0.01	0.02 ± 0.01	0.013 ± 0.008	0.009 ± 0.009	0.012 ± 0.009
extreme bias (left)	0.09 ± 0.02	0.012 ± 0.009	0.04 ± 0.01	0.02 ± 0.01	0.00 ± 0.01

↙	right leaning (no aggr.)	center	left leaning	left	extreme bias (left)
pro-Clinton	0.006 ± 0.006	0.046 ± 0.007	0.065 ± 0.008	0.022 ± 0.009	0.006 ± 0.006
pro-Trump	0.002 ± 0.001	0.032 ± 0.007	0.015 ± 0.003	0.025 ± 0.007	0.013 ± 0.006
fake news (no aggr.)	0.05 ± 0.01	0.039 ± 0.009	0.013 ± 0.002	0.025 ± 0.008	0.028 ± 0.009
extreme bias (right) (no aggr.)	0.06 ± 0.01	0.041 ± 0.009	0.017 ± 0.002	0.004 ± 0.001	0.030 ± 0.010
right	0.08 ± 0.01	0.042 ± 0.009	0.018 ± 0.003	0.028 ± 0.009	0.034 ± 0.009
right leaning (no aggr.)	0.29 ± 0.01	0.036 ± 0.010	0.03 ± 0.01	0.016 ± 0.008	0.0022 ± 0.0009
center	0.005 ± 0.007	0.267 ± 0.009	0.18 ± 0.01	0.020 ± 0.009	0.021 ± 0.008
left leaning	0.002 ± 0.002	0.18 ± 0.01	0.300 ± 0.009	0.013 ± 0.008	0.013 ± 0.007
left	0.021 ± 0.008	0.07 ± 0.01	0.10 ± 0.01	0.162 ± 0.010	0.07 ± 0.01
extreme bias (left)	0.010 ± 0.010	0.024 ± 0.004	0.05 ± 0.01	0.03 ± 0.01	0.26 ± 0.01

Supplementary Table 13: Maximum causal effect without news aggregators. Maximum causal effect values (± s.d.) between the activity of the top 100 spreaders of each media category, where news aggregators websites have removed, and the activity of the presidential candidate supporters. We see that our conclusions stay valid even without the news aggregators, namely the domination of center and left leaning influencers in term of causal effects. We observe a small decrease in the intensity of the causal effect of center influencers toward Clinton supporters (0.065 to 0.046), but the effect is still the second most important after the left leaning influencers. We also observe a small increase of the causal effect of Clinton supporters on the fake news top spreaders. Without the news aggregators, the top fake news, extreme bias (right) and right leaning spreaders have a smaller causal effect on the other groups.

client name	number of tweets with a URL
Twitter for iPhone	14 215 411
Twitter Web Client	13 045 560
Twitter for Android	10 192 781
Twitter for iPad	3 355 197
Facebook	1 254 619
TweetDeck	1 079 637
Mobile Web (M5)	951 749
Mobile Web	452 812
Google	410 514
Twitter for Windows	200 088
Twitter for Windows Phone	170 529
Mobile Web (M2)	161 682
Twitter for BlackBerry	93 937
iOS	72 334
Twitter for Android Tablets	56 007
Twitter for Mac	43 993
OS X	40 642
Twitter for BlackBerry®	25 140

Supplementary Table 14: List of Twitter official clients. We also display the number of tweets containing a URL and originating from each official client. The number of tweets with a URL originating from official clients represent 82% of the total number of tweets with a URL.

$$\mathcal{P}_0 =(0_{t-1}, 1_{t-1}, 8_{t-1}, 7_{t-1}, 6_{t-1}, 9_{t-1}, 0_{t-2}, 1_{t-2}, 8_{t-2}, 6_{t-2}, 7_{t-2}, 0_{t-3}, 1_{t-3}, 8_{t-3}, 3_{t-3}, 2_{t-3}, 0_{t-4}, 1_{t-4}, 8_{t-4}, 0_{t-5}, 1_{t-5}, 8_{t-5}, 0_{t-6}, 1_{t-6}, 8_{t-6}, 0_{t-7}, 1_{t-7}, 8_{t-7}, 0_{t-8}, 1_{t-8}, 8_{t-8}, 4_{t-8}, 0_{t-9}, 1_{t-9}, 8_{t-9}, 6_{t-9}, 4_{t-9}, 0_{t-10}, 1_{t-10}, 6_{t-10}, 0_{t-11}, 1_{t-11}, 8_{t-11}, 0_{t-12}, 9_{t-12}, 1_{t-12}, 6_{t-12}, 0_{t-13}, 8_{t-13}, 1_{t-13}, 0_{t-14}, 1_{t-14}, 0_{t-15}, 0_{t-16}, 0_{t-17}, 1_{t-17}, 0_{t-18}, 1_{t-18})$$

$$\mathcal{P}_1 =(1_{t-1}, 0_{t-1}, 9_{t-1}, 1_{t-2}, 0_{t-2}, 2_{t-2}, 6_{t-2}, 1_{t-3}, 0_{t-3}, 2_{t-3}, 8_{t-3}, 1_{t-4}, 0_{t-4}, 1_{t-5}, 0_{t-5}, 2_{t-5}, 8_{t-5}, 1_{t-6}, 0_{t-6}, 1_{t-7}, 0_{t-7}, 1_{t-8}, 0_{t-8}, 1_{t-9}, 0_{t-9}, 1_{t-10}, 0_{t-10}, 1_{t-11}, 8_{t-11}, 0_{t-11}, 1_{t-12}, 7_{t-12}, 0_{t-12}, 1_{t-13}, 8_{t-13}, 0_{t-13}, 1_{t-14}, 2_{t-14}, 1_{t-15}, 0_{t-15}, 0_{t-16}, 7_{t-16}, 6_{t-17}, 1_{t-17}, 0_{t-17}, 0_{t-18}, 1_{t-18})$$

$$\mathcal{P}_2 =(2_{t-1}, 5_{t-1}, 9_{t-1}, 3_{t-1}, 4_{t-1}, 6_{t-1}, 1_{t-1}, 2_{t-2}, 3_{t-2}, 9_{t-2}, 6_{t-2}, 1_{t-2}, 5_{t-2}, 2_{t-3}, 4_{t-3}, 1_{t-3}, 6_{t-3}, 3_{t-3}, 2_{t-4}, 8_{t-4}, 1_{t-4}, 6_{t-5}, 5_{t-5}, 2_{t-5}, 2_{t-6}, 1_{t-6}, 5_{t-7}, 2_{t-8}, 8_{t-9}, 1_{t-9}, 6_{t-11}, 6_{t-13}, 2_{t-13})$$

$$\mathcal{P}_3 =(3_{t-1}, 5_{t-1}, 2_{t-1}, 4_{t-1}, 9_{t-1}, 6_{t-1}, 0_{t-1}, 3_{t-2}, 6_{t-2}, 5_{t-2}, 9_{t-2}, 2_{t-2}, 4_{t-2}, 4_{t-3}, 4_{t-4}, 0_{t-4}, 5_{t-5}, 6_{t-5}, 8_{t-6}, 0_{t-7}, 4_{t-7}, 6_{t-11}, 6_{t-13}, 3_{t-17}, 5_{t-18})$$

$$\mathcal{P}_4 =(4_{t-1}, 5_{t-1}, 2_{t-1}, 3_{t-1}, 6_{t-1}, 9_{t-1}, 7_{t-1}, 4_{t-2}, 5_{t-2}, 3_{t-2}, 2_{t-2}, 4_{t-3}, 2_{t-3}, 4_{t-4}, 6_{t-5}, 4_{t-5}, 5_{t-5}, 1_{t-5}, 3_{t-6}, 1_{t-8}, 2_{t-13}, 3_{t-17})$$

$$\mathcal{P}_5 =(5_{t-1}, 4_{t-1}, 2_{t-1}, 3_{t-1}, 7_{t-1}, 6_{t-1}, 5_{t-2}, 6_{t-2}, 2_{t-2}, 1_{t-2}, 3_{t-2}, 4_{t-3}, 5_{t-4}, 7_{t-4}, 6_{t-5}, 5_{t-5}, 2_{t-5}, 4_{t-6}, 0_{t-18})$$

$$\mathcal{P}_6 =(6_{t-1}, 7_{t-1}, 8_{t-1}, 0_{t-1}, 5_{t-1}, 1_{t-1}, 6_{t-2}, 7_{t-2}, 8_{t-2}, 9_{t-2}, 2_{t-2}, 6_{t-3}, 7_{t-3}, 8_{t-3}, 2_{t-3}, 1_{t-3}, 6_{t-4}, 7_{t-4}, 8_{t-4}, 5_{t-4}, 6_{t-5}, 8_{t-5}, 4_{t-5}, 7_{t-5}, 6_{t-6}, 7_{t-6}, 8_{t-6}, 9_{t-6}, 5_{t-6}, 2_{t-6}, 6_{t-7}, 7_{t-7}, 7_{t-8}, 6_{t-8}, 2_{t-9}, 6_{t-10}, 8_{t-18})$$

$$\mathcal{P}_7 =(7_{t-1}, 6_{t-1}, 8_{t-1}, 0_{t-1}, 7_{t-2}, 6_{t-2}, 8_{t-2}, 6_{t-3}, 7_{t-3}, 8_{t-3}, 7_{t-4}, 6_{t-4}, 8_{t-4}, 7_{t-5}, 6_{t-5}, 4_{t-5}, 8_{t-5}, 8_{t-6}, 6_{t-6}, 7_{t-6}, 6_{t-7}, 6_{t-8}, 6_{t-9}, 6_{t-10}, 7_{t-11}, 6_{t-17})$$

$$\mathcal{P}_8 =(8_{t-1}, 9_{t-1}, 7_{t-1}, 6_{t-1}, 8_{t-2}, 6_{t-2}, 7_{t-2}, 4_{t-2}, 5_{t-2}, 8_{t-3}, 6_{t-3}, 7_{t-3}, 9_{t-3}, 2_{t-3}, 0_{t-3}, 1_{t-3}, 7_{t-4}, 8_{t-4}, 6_{t-4}, 9_{t-4}, 0_{t-4}, 7_{t-5}, 8_{t-5}, 2_{t-5}, 8_{t-6}, 5_{t-6}, 7_{t-6}, 6_{t-6}, 0_{t-6}, 7_{t-7}, 8_{t-7}, 9_{t-7}, 1_{t-7}, 6_{t-8}, 8_{t-8}, 8_{t-9}, 7_{t-11}, 6_{t-13})$$

$$\mathcal{P}_9 =(9_{t-1}, 8_{t-1}, 7_{t-1}, 5_{t-1}, 1_{t-1}, 0_{t-1}, 9_{t-2}, 8_{t-2}, 2_{t-2}, 6_{t-2}, 1_{t-2}, 9_{t-3}, 8_{t-3}, 2_{t-3}, 9_{t-4}, 8_{t-4}, 0_{t-4}, 2_{t-5}, 8_{t-5}, 3_{t-5}, 5_{t-5}, 3_{t-6}, 9_{t-6}, 0_{t-6}, 8_{t-7}, 7_{t-7}, 0_{t-7}, 9_{t-8}, 6_{t-8}, 3_{t-8}, 0_{t-8}, 7_{t-11}, 1_{t-11}, 9_{t-12}, 3_{t-13}, 9_{t-13}, 2_{t-13}, 6_{t-14}, 0_{t-14})$$

Supplementary Table 15: Parents $\mathcal{P}$ for each time series estimated with the causal discovery algorithm. 0 stands for pro-Clinton, 1 for pro-Trump, 2 for top fake news spreaders, 3 for top extreme bias (right) spreaders, 4 for top right spreaders, 5 for top right leaning spreaders, 6 for top center spreaders, 7 for top left leaning spreaders, 8 for top left spreaders and 9 for top extreme bias (left) spreaders.

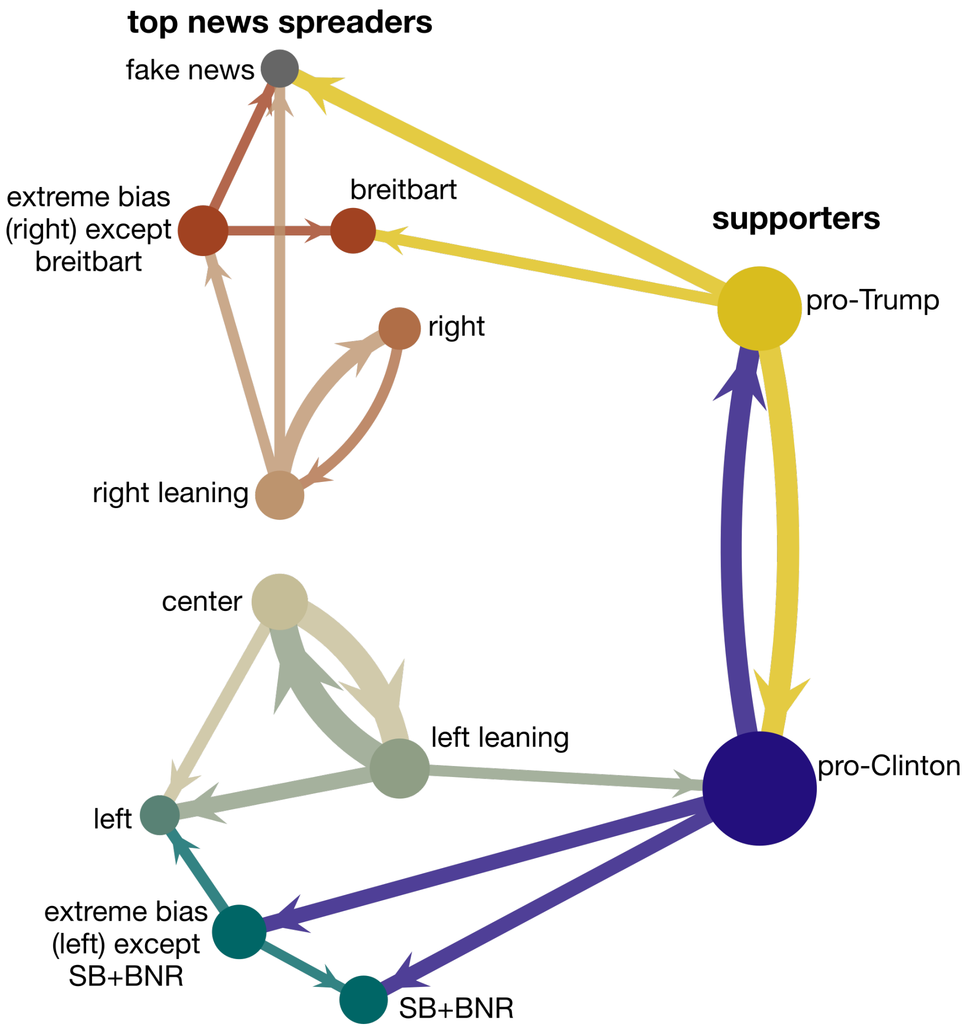

Supplementary Figure 1: Causal graph obtained when considering breitbart and shareblue+bluenationreview (SB+BNR) as separated from extreme bias (right) and extreme bias (left), respectively. We only show causal effects larger than 0.05.

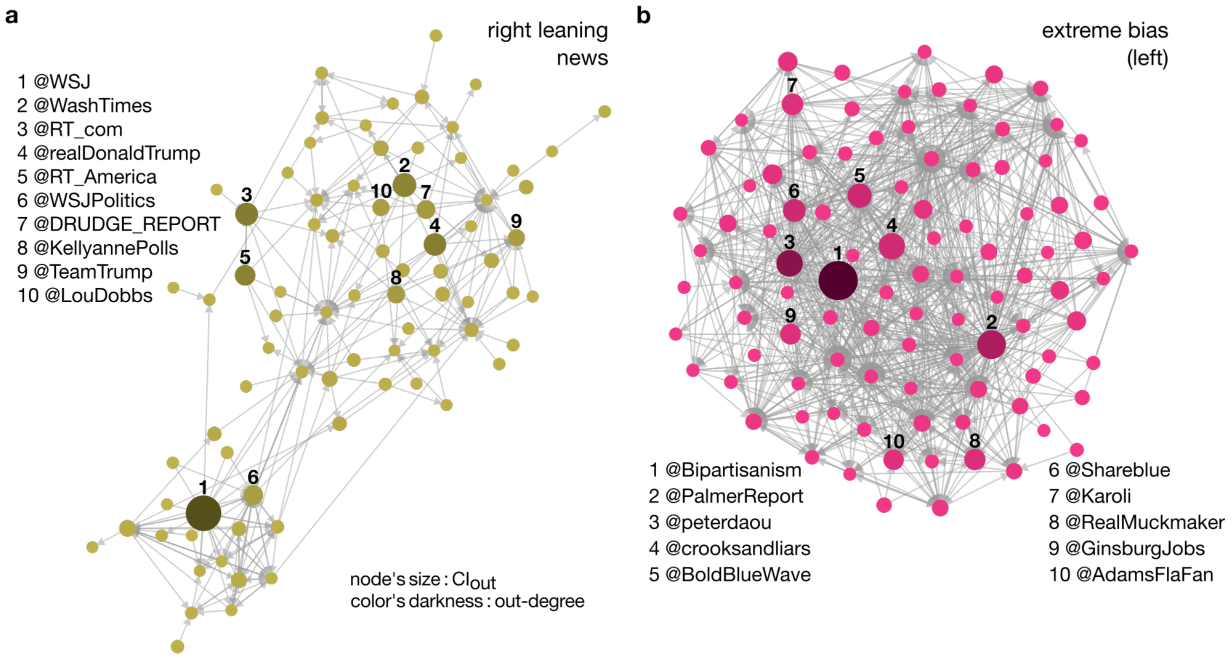

Supplementary Figure 2: Retweet networks formed by the top 100 influencers of right leaning (a) and extreme bias (left) news (b). The direction of the links represents the flow of information between users. The size of the nodes is proportional to their $\mathrm{CI}_{\mathrm{out}}$ values and the shade of the nodes' color represents their out-degree from dark (high out-degree) to light (low out-degree).

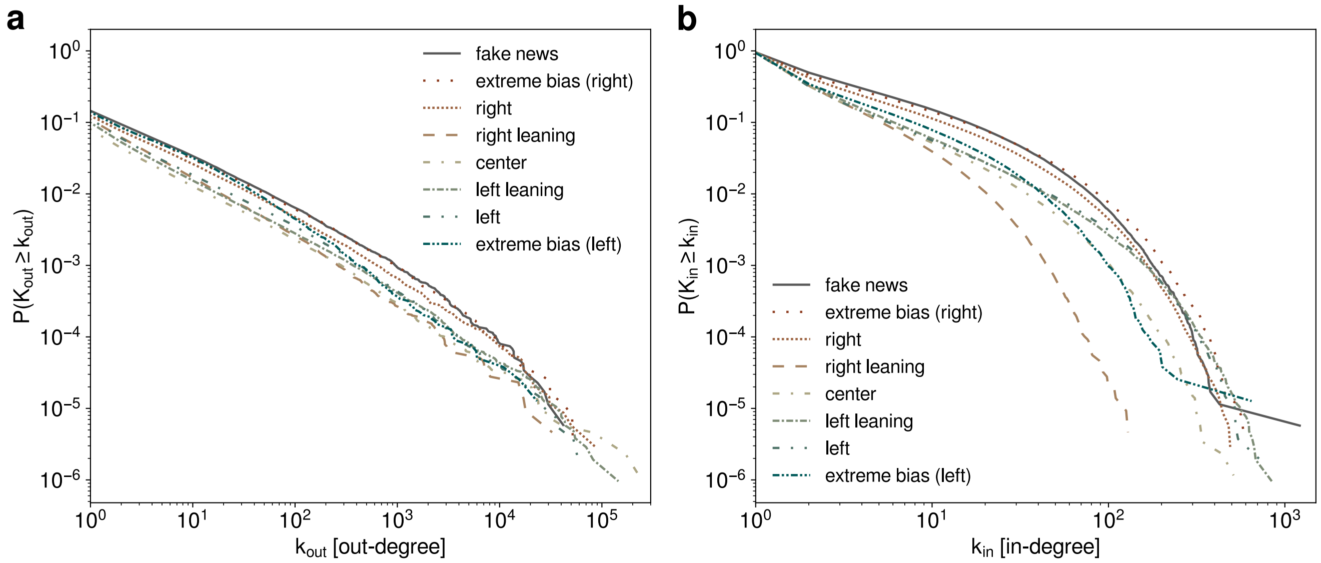

Supplementary Figure 3: Empirical complementary cumulative distribution function (CCDF) of the out-degree (a) and in-degree (b) of the retweet networks for each media category. The CCDF, $P(K \geq k)$, gives the probability that the in- (or out-) degree of a node is greater of equal to k. The out-degree of a node, i.e. a user, is equal to the number of different users that have retweeted at least one of her/his tweets with a URL directing to a news outlet. Its in-degree represents the number of different users she/he retweeted. The CCDF of the fake, extremely biased (right) and right networks are characterized by less steep slopes on the log-log plots than the other distributions, resulting in a larger average degree, thus indicating a wider diversity of attention from the audience of these news, i.e. they typically retweet more people and are retweeted by more people, than the audience of more traditional news.

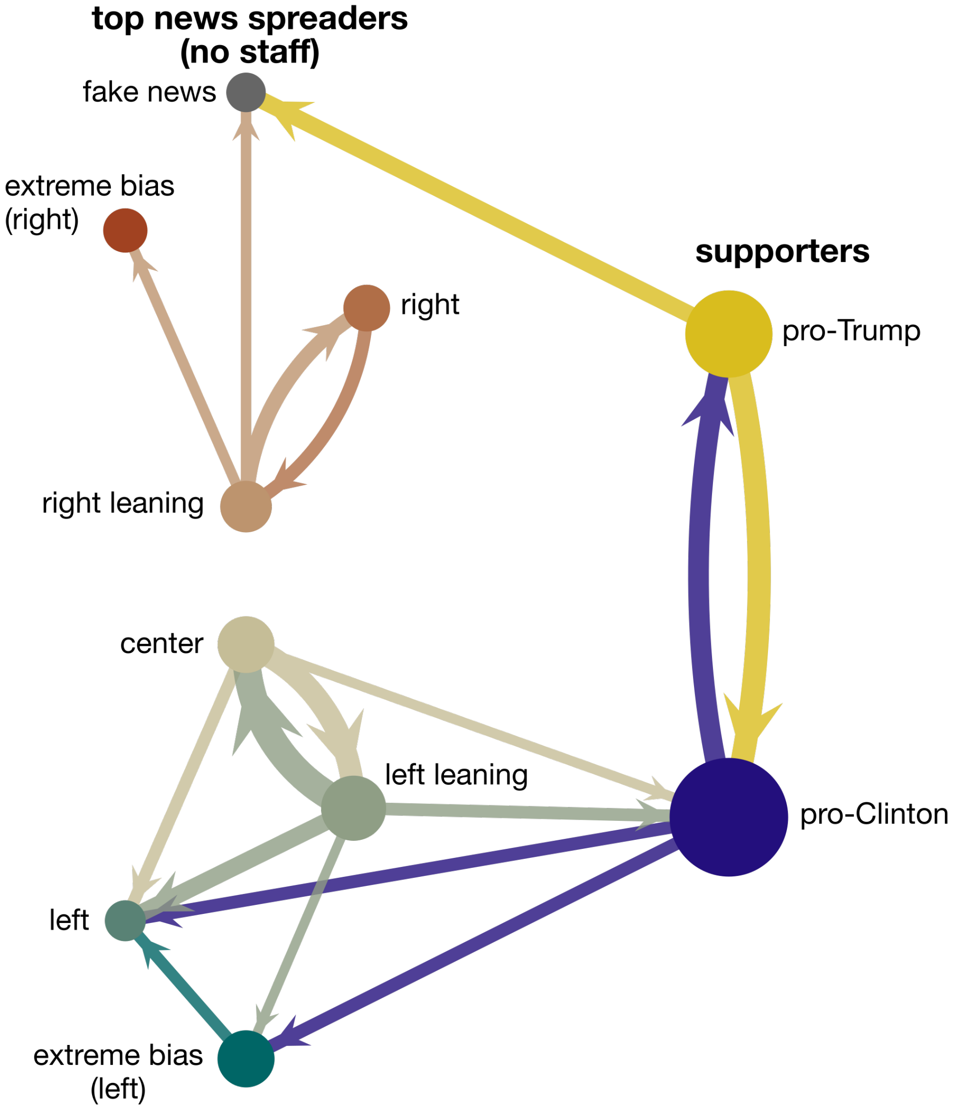

Supplementary Figure 4: Causal graph obtained after removing all users linked to the campaign staff of each candidate from the influencers. We only show causal effects larger than 0.05.

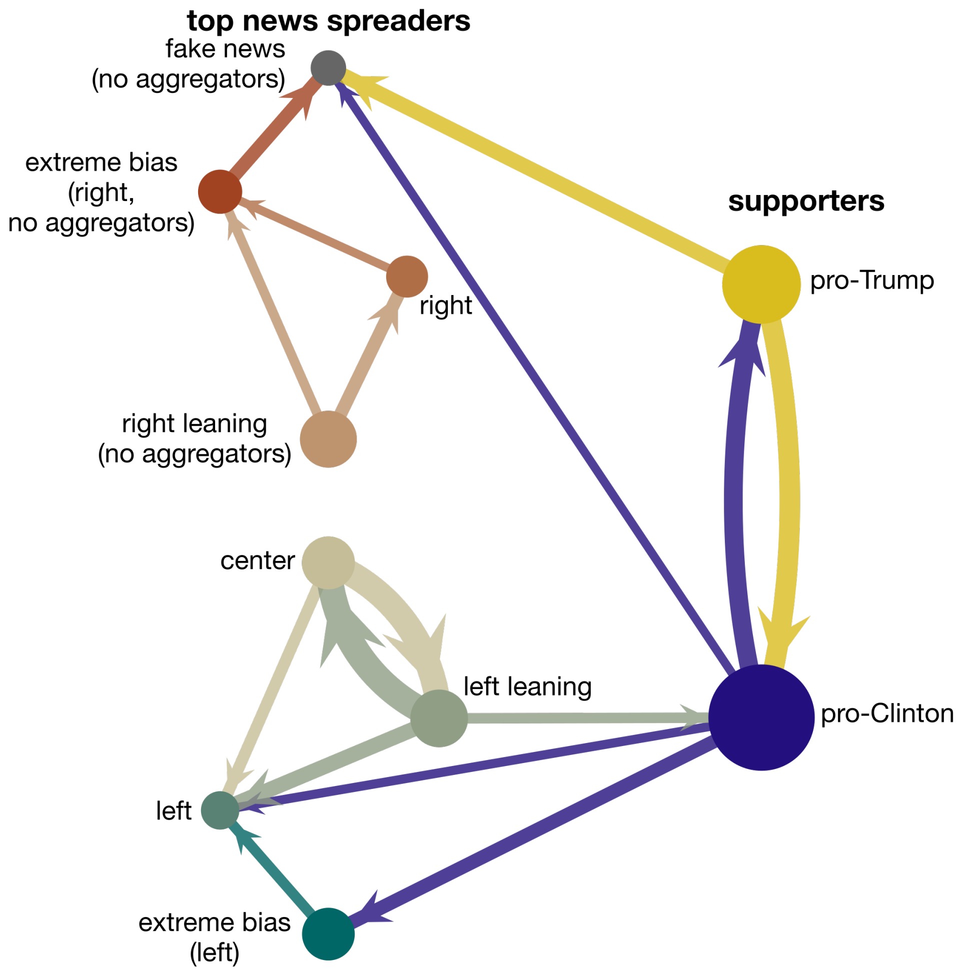

Supplementary Figure 5: Causal graph obtained after removing news aggregators websites. We only show causal effects larger than 0.05.

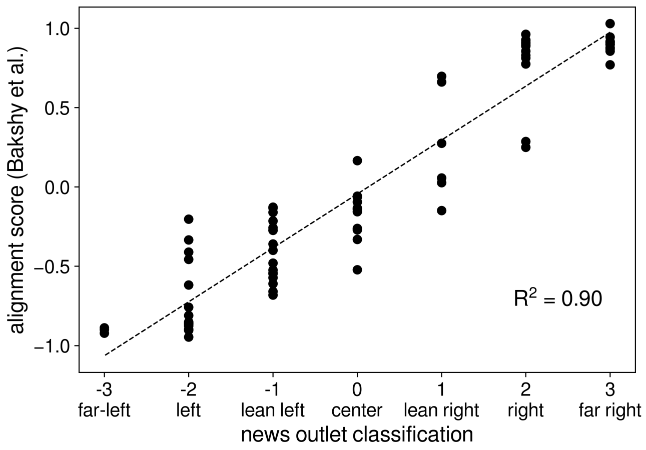

Supplementary Figure 6: Comparison of the news outlet political alignment we obtained with the results of [1].

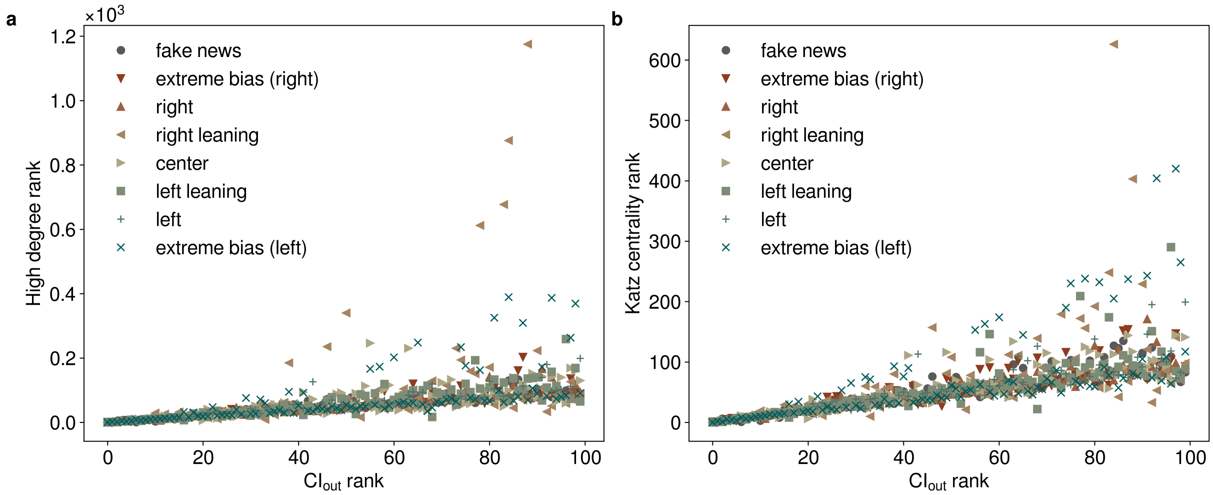

Supplementary Figure 7: Comparison of Collective Influence super-spreader ranking ($\mathbf{CI_{out}}$) with High degree ranking (a) and Katz centrality ranking (b).

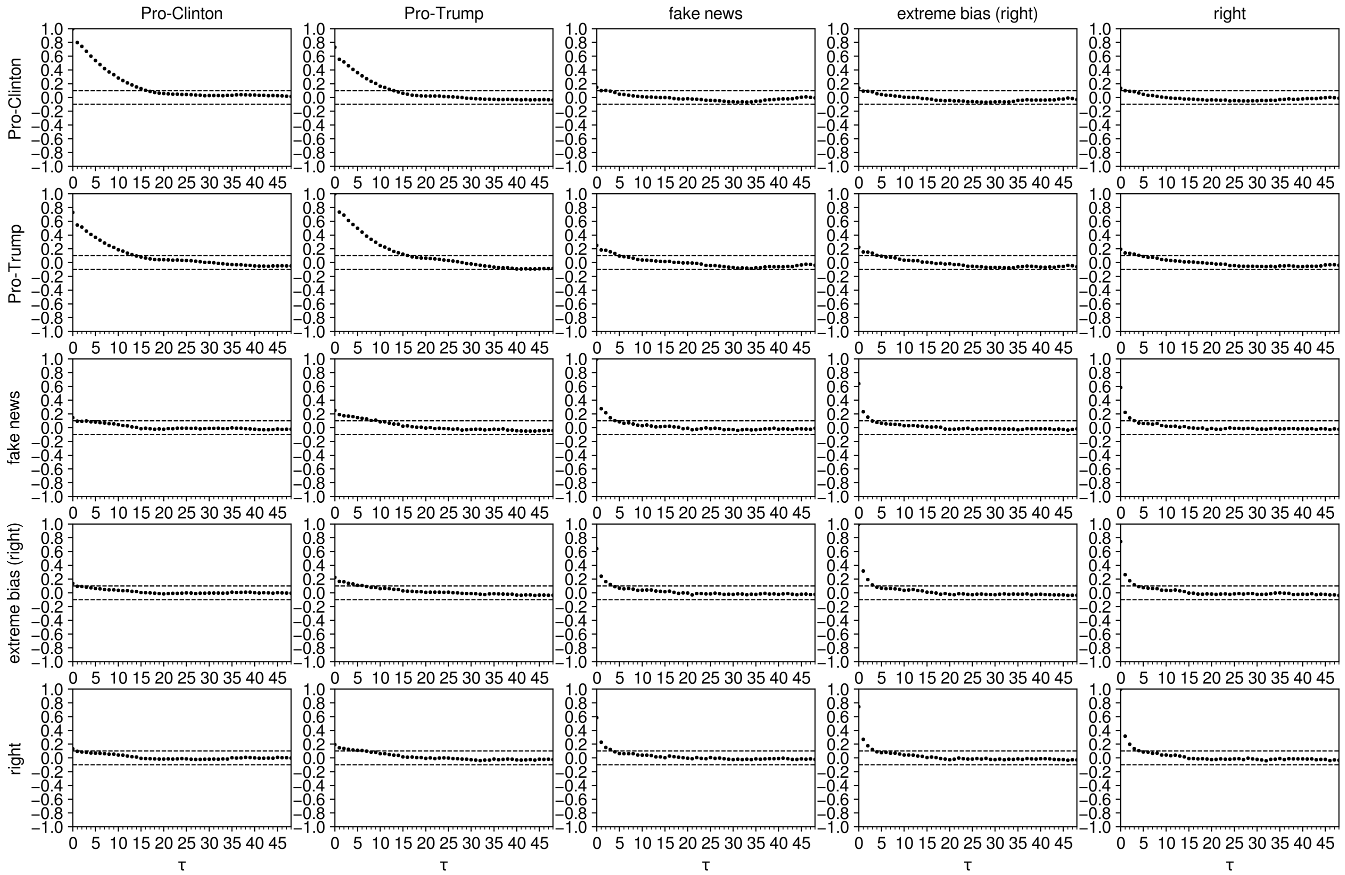

Supplementary Figure 8: Pairwise lagged correlations between the activity time series of top 100 influencers of fake, extreme bias (right) and right news as well as and Trump and Clinton supporters. The time lag, τ, is expressed in data time points corresponding to 15 min interval. The horizontal dashed line represents a correlation value of 0.1 and -0.1.

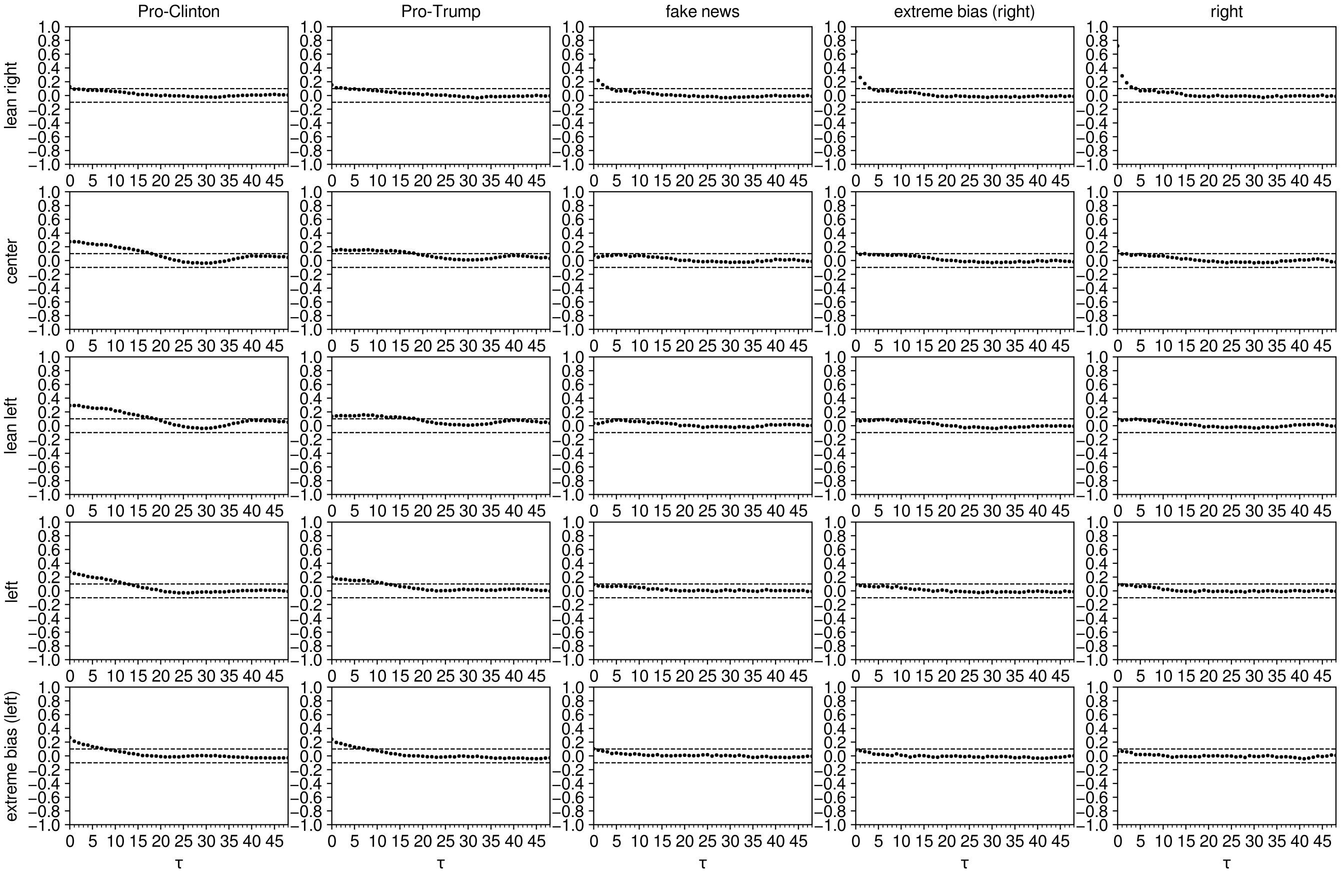

Supplementary Figure 9: Pairwise lagged correlations between the activity time series of top 100 influencers of fake, extreme bias (right), right leaning, center, left leaning, left and extreme bias (left) news as well as and Trump and Clinton supporters. The time lag, τ, is expressed in data time points corresponding to 15 min interval. The horizontal dashed line represents a correlation value of 0.1 and -0.1.

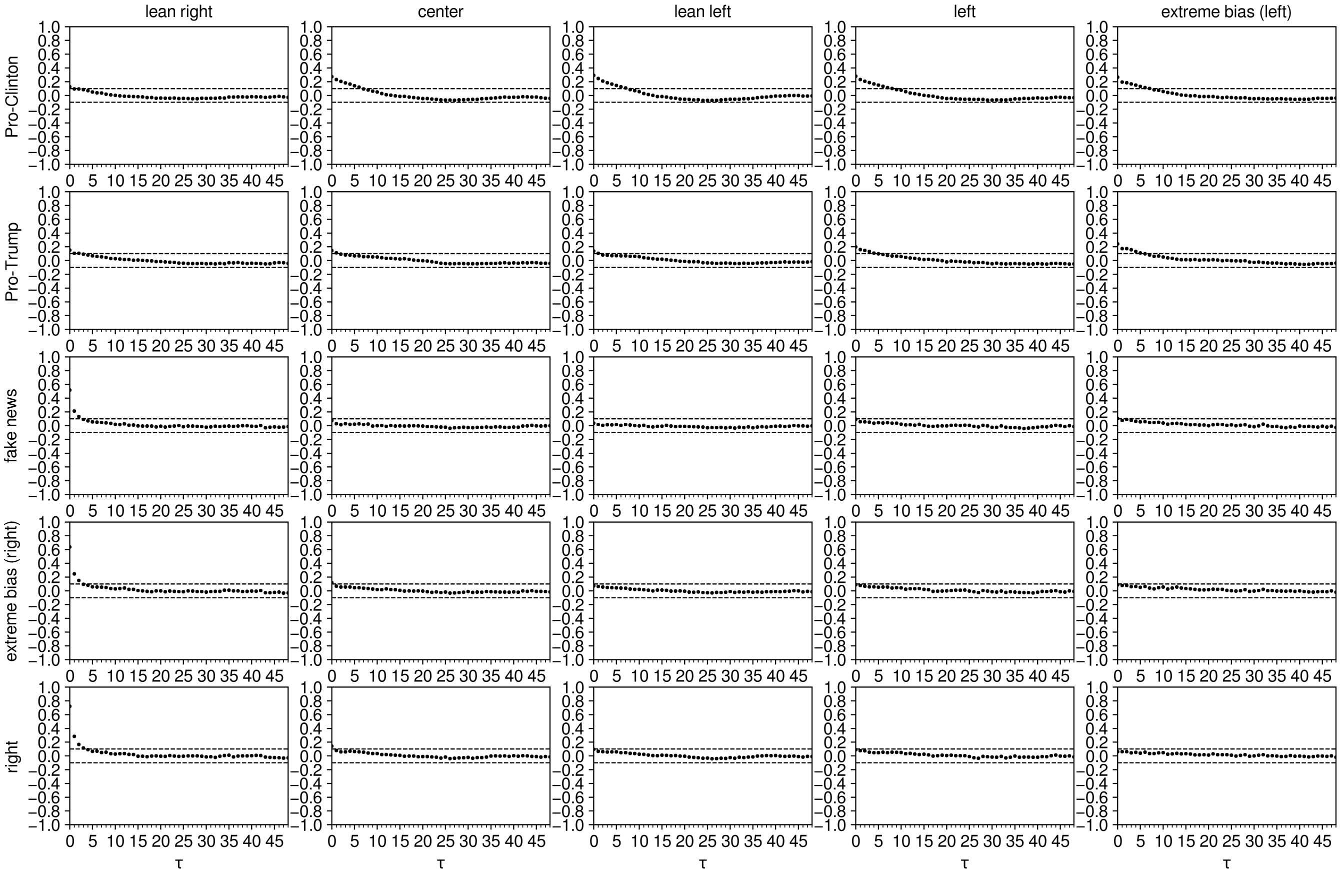

Supplementary Figure 10: Pairwise lagged correlations between the activity time series of top 100 influencers of fake, extreme bias (right), right leaning, center, left leaning, left and extreme bias (left) news as well as and Trump and Clinton supporters. The time lag, τ, is expressed in data time points corresponding to 15 min interval. The horizontal dashed line represents a correlation value of 0.1 and -0.1.

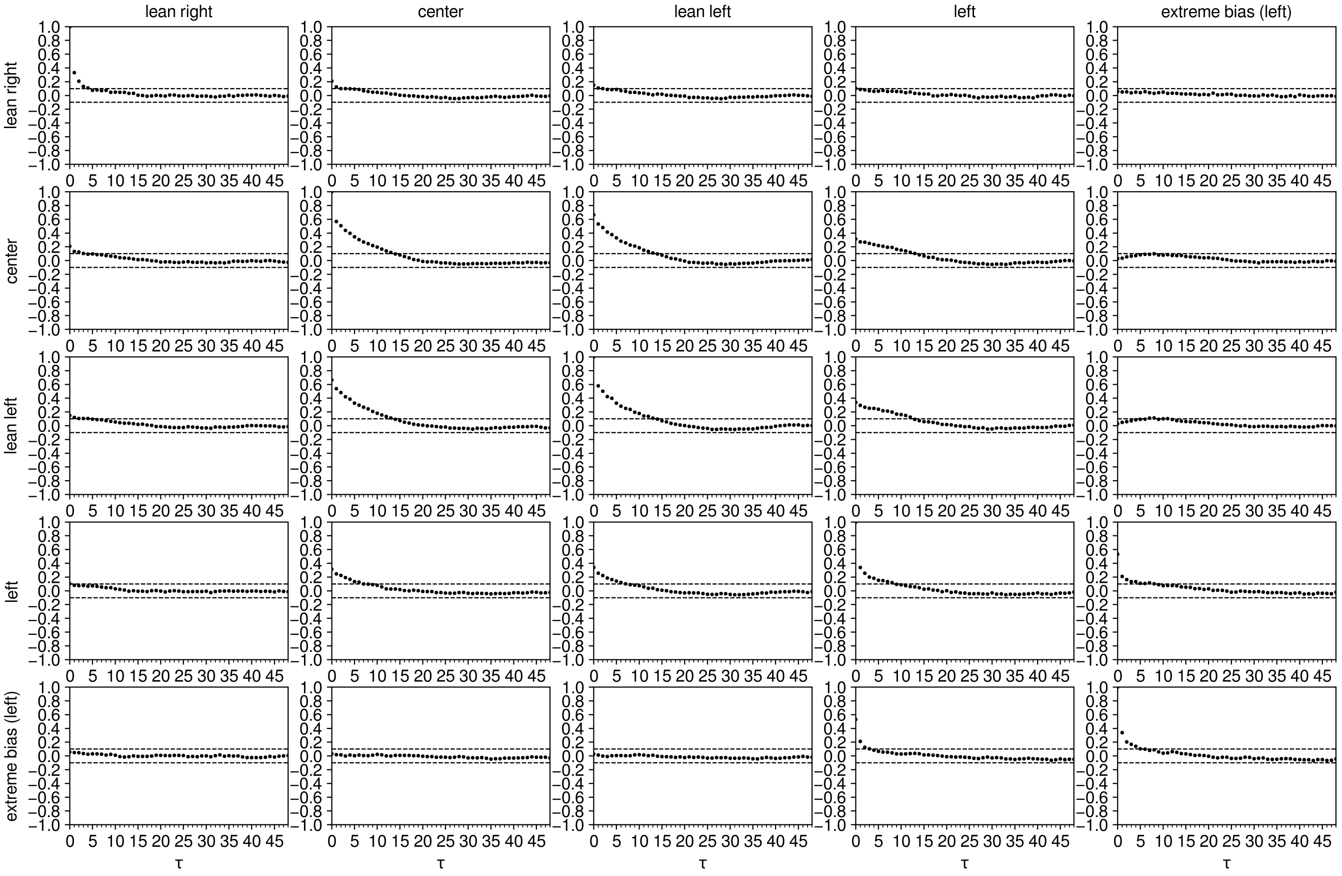

Supplementary Figure 11: Pairwise lagged correlations between the activity time series of top 100 influencers of right leaning, center, left leaning, left and extreme bias (left) news. The time lag, τ, is expressed in data time points corresponding to 15 min interval. The horizontal dashed lines represents a correlation value of 0.1 and -0.1.

Supplementary References

[1] Bakshy, E., Messing, S. & Adamic, L. A. Exposure to ideologically diverse news and opinion on Facebook. *Science* **348**, 1130–1132 (2015). http://www.sciencemag.org/cgi/doi/10.1126/science.aaa1160.
